# IL-6 signaling is required for the development and regeneration of ear cartilage in microtia

**DOI:** 10.3389/fcell.2025.1625058

**Published:** 2025-07-30

**Authors:** Wenkang Luan, Shujun Fan, Hanyi Jiang, Dongwen Jiang, Jinxiu Yang, Leren He

**Affiliations:** ^1^Department of Auricular Reconstruction, Plastic Surgery Hospital, Chinese Academy of Medical Sciences and Peking Union Medical College, Beijing, China; ^2^Center for Cleft Lip and Palate Treatment, Plastic Surgery Hospital, Chinese Academy of Medical Sciences and Peking Union Medical College, Beijing, China

**Keywords:** microtia, IL-6, intercellular communication, cartilage microspheres, development and regeneration of auricular cartilage

## Abstract

**Introduction:**

Congenital microtia is a birth defect characterized by auricular underdevelopment, with unclear pathogenesis and unidentified pathogenic genes.

**Methods:**

Differential expression analysis, weighted co-expression network analysis (WGCNA), protein-protein interaction (PPI) networks and support vector machine recursive feature elimination (SVM-RFE) identified the key biomolecules in microtia. Single-cell and intercellular communication analysis were used to decipher the key intercellular signaling pathway. We extracted primary cells and conducted Immuno precipitation mass spectrometry (IP-MS), co-Immuno precipitation (Co-IP) and RNA-sequencing (RNA-seq) to confirmed the mechanism. The intercellular communication network was confirmed through the cell co-culture system. Organoid and animal models further validated the role of key biomolecules.

**Results:**

We found that IL-6 may be the key biomolecule in microtia. Normal ear cartilage tissue is mainly composed of chondrocytes, but microtia auricular ear tissue contained chondrocytes and stem cells. IL-6 signaling pathway is the main intercellular communication pathways in microtia. We extracted primary chondrocytes and stem cells, and proved that IL-6 promotes the growth and migration of primary cells. The binding of IL-6 and IL-6R and Glycoprotein 130 (GP130) and the activation of their downstream were confirmed. Furthermore, IL-6 signaling pathway was proved to involve in the intercellular communication of microtia. Cartilage microspheres demonstrated the role of IL-6 in regeneration of ear cartilage. The preventive intervention of adeno-associated virus (AAV) on pregnant mice confirmed the role of IL-6 *in vivo*.

**Conclusion:**

IL-6 signaling is the key biomolecule in the development and regeneration of auricular cartilage in microtia. IL-6 is a potential biomarker and preventive and therapeutic target for microtia patients.

## Introduction

Congenital microtia is a birth defect characterized by underdevelopment of the auricle, often accompanied by impaired hearing function and developmental disorders of tissues and organs, such as the craniofacial region (Sun et al., 2023; Zhang et al., 2018). The incidence rate of microtia is about 0.8–17.4/10,000, ranking second among maxillofacial malformations (Joukhadar et al., 2020; Luquetti et al., 2011). It not only affects the facial appearance of children but also causes psychological and social behavioral problems. Currently, auricle reconstruction surgery using rib cartilage as the scaffold is the main treatment method for microtia (Bly et al., 2016). However, in addition to the risk of surgery, surgery can also cause pain, scars, and clicking sensation of the chest and chest contour deformity in patients (Uppal et al., 2008). Thus, there is an urgent need to further study the pathogenesis of microtia.

Extensive studies have been devoted to exploring the mechanisms underlying the occurrence of microtia (Lu et al., 2020), its pathogenesis remains unclear, and no clear pathogenic genes have been discovered. The difference in the severity of the microtia lies in the degree of auricular cartilage tissue development. Therefore, it is necessary to explore the key genes and signaling pathways involved in the development of auricular cartilage in microtia. In this study, based on the transcriptome data of the samples we collected, we identified IL-6 and CXCL1 as the only two hub genes simultaneously recognized as diagnostic biomarkers for microtia through differential expression analysis, weighted Gene Co-expression Network Analysis (WGCNA), support Vector Machine-Recursive Feature Elimination (SVM-RFE), and protein-Protein Interaction (PPI) network analysis.

Single-cell sequencing can identify genetic information at the single-cell level, thereby reflecting the essence of disease development (Aran et al., 2019; Stubbington et al., 2017). To deepen our understanding of microtia, we analyzed single-cell transcriptome data from the GEO#GSE179135 datasets. We found that normal auricular cartilage tissue is mainly composed of chondrocytes, whereas microtia auricular cartilage tissue is composed of chondrocytes and tissue stem cells. Intercellular communication mediated by cytokines and their receptors is closely related to various biological processes such as cell differentiation and organ development (Huang et al., 2021; Tsang et al., 2017). We conducted intercellular communication analysis and found that the IL-6 signaling pathway is one of the main intercellular communication pathways between stem cells and chondrocytes in microtia.

IL-6 is a multifunctional cytokine with multiple proinflammatory and anti-inflammatory (Scheller et al., 2011), the role of IL-6 in microtia has not yet been studied. Here, we identified significant IL-6 under-expression in microtia ear cartilage tissue. We further confirmed that IL-6 promotes the growth, migration, and development of primary chondrocytes and primary stem cells derived from ear cartilage (PSCECs). Additionally, IL-6 and its receptors (IL-6R, GP130/IL-6ST) bind to these cells. A co-culture system demonstrated that the IL-6 pathway mediates communication between chondrocytes and PSCECs. Organoid and animal models further validated the role of IL-6. In summary, these results suggest that IL-6 is a biomarker and potential therapeutic target for microtia.

## Results

### Identification of the key genes in microtia

**FIGURE 1:**
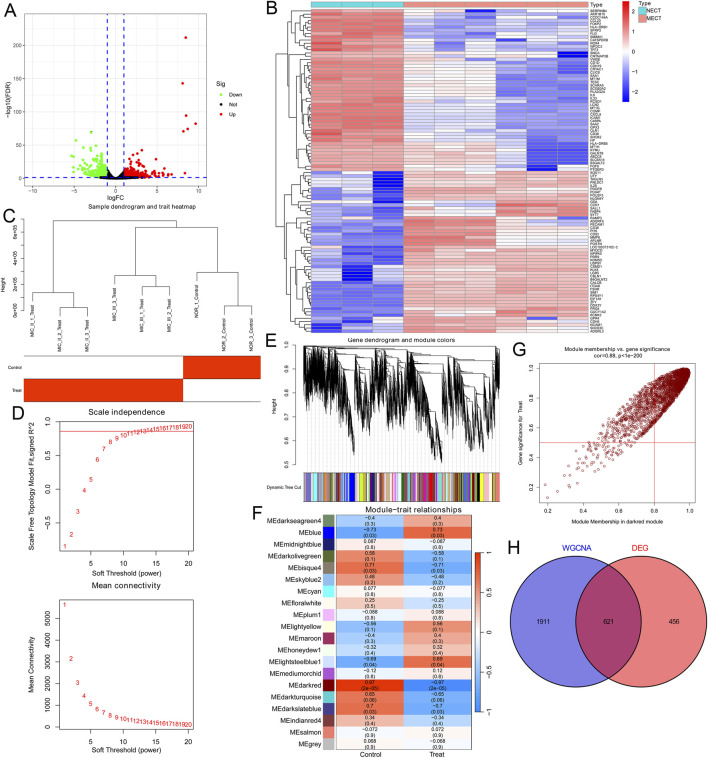
Identification of the key genes in microtia. **(A)** Volcano plot showed all DEGs over 2.0-fold change between 3 NECT and 6 MECT. Upregulated genes were marked in red and downregulated genes were marked in green. **(B)** The cluster heat map showed the expression of DEGs in the top 50 and bottom 50. **(C)** The sample clustering tree of 3 NECT and 6 MECT. **(D)** Analysis of the scale-free index for various soft-threshold power and the mean connectivity for various soft-threshold powers. **(E)** Dendrogram of genes clustered based on the measurement of dissimilarity. The colour band shows the results of identifying modules and merging similar modules. **(F)** Heatmap of the correlation between the module eigengenes and microtia. **(G)** Analysis of gene significance for microtia and module membership in dark red module. **(H)** Venn diagram showed the intersection of the DEGs in microtia and the microtia-associated genes in dark red module.

We first analyzed the genomic profiles of three normal ear cartilage tissues (NECT) and six microtia ear cartilage tissues (MECT) using RNA-seq to identify DEGs in the microtia. 1,077 genes (including 518 upregulated genes and 559 downregulated genes) that changed more than 2.0-fold between NECT and MECT were found (Supplementary Table S1). All DEGs are shown in the volcano plot (Figure 1A), and the cluster heat map displays the 50 upregulated genes and 50 downregulated genes with the most significant differences (Figure 1B). To identify the microtia-associated genes, we performed WGCNA analysis based on the transcriptome data of the samples, and 13,975 genes were included after normalization of the data. We drew a sample clustering tree after clustering the samples (Figure 1C). To construct a scale-free network, the soft threshold was set to 13 (*R* ^2^ = 0.861, truncated *R* ^2^ = 0.961, slope = −1.025) (Figure 1D). Twenty modules and their corresponding genes were identified (Figure 1E). The dark red module was found to be most relevant to microtia (Figures 1F,G); thus, 2,532 genes in this module (Supplementary Table S2) were considered to be microtia-associated genes. Finally, we considered the intersection of the DEGs and microtia-associated genes, resulting in 621 key genes in the microtia (Figure 1H; Supplementary Table S3).

### Identifying specific biomarkers in microtia

**FIGURE 2:**
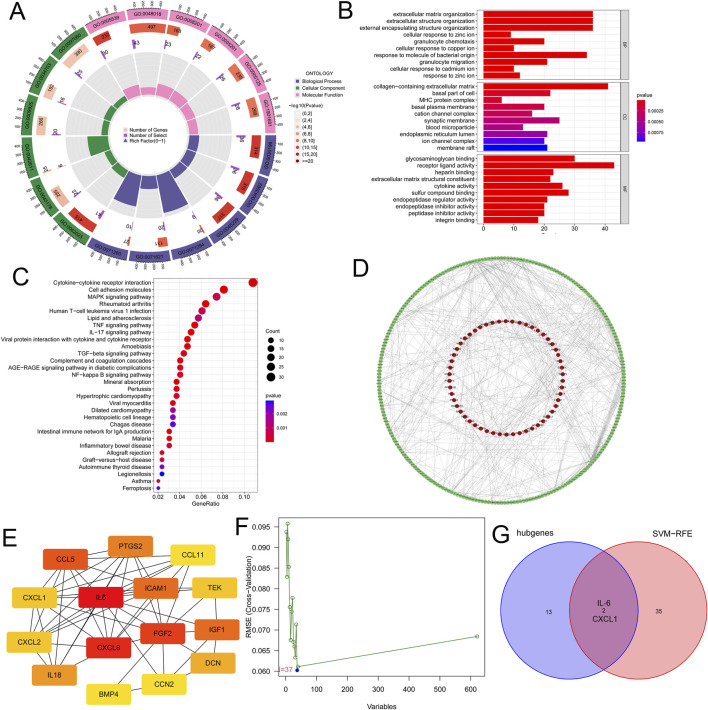
Identifying specific biomarkers in microtia. **(A)** GO enrichment analysis of the 621 key genes. BP, CC and MF were displayed in different colours respectively. All genes and the number of key genes of microtia involved in different cell functions were also displayed. **(B)** GO enrichment analysis of the 621 key genes, and we showed the top ten cell functions of BP, CC and MF involved in these key genes. **(C)** KEGG enrichment analysis of the 621 key genes showcased the signaling pathways involved in these key genes. **(D)** The PPI network of the 621 key genes were constructed by using STRING website and Cytoscape software. **(E)** CytoHubba plugin in Cytoscape software identified the 15 the network hubgenes, the network diagram of these hubgenes was shown. **(F)** SVM-RFE was used to screen out the potential diagnostic biomarkers of microtia among 621 key genes, and 37 genes were identified as diagnostic biomarkers for microtia. **(G)** Venn diagram showed the intersection of the results of hubgenes and SVM-RFE, that was IL-6 and CXCL1.

We conducted an enrichment analysis of GO and KEGG on the 621 key microtia genes. In the biological process (BP) group, genes were mainly enriched in extracellular structure organization and extracellular matrix organization, whereas in the component (CC) group, genes were mainly related to collagen-containing extracellular matrix and basal part of the cell; in the molecular function (MF) group, genes were mainly associated with receptor ligand activity and cytokine activity (Figures 2A,B). KEGG analysis showed that the key genes were enriched in cytokine-cytokine receptor interactions (Figure 2C). This suggests that cytokine and intercellular communication may play important roles in microtia.

Next, we built a PPI network of 621 key genes using the STRING website (https://cn.string-db.org/) and the Cytoscape software (Figure 2D; Supplementary Table S4). We further scored each gene to search for hub genes in the network using the cytoHubba plugin of the Cytoscape software (Supplementary Table S5). The top 15 genes with the highest scores (the number of neighboring nodes) were selected as hub genes (Supplementary Table S6), and the network diagram of these hub genes is shown in Figure 2E. Moreover, we used a machine learning algorithm (SVM-RFE) to screen the potential diagnostic biomarkers of microtia among the 621 key genes, and 37 genes were identified as diagnostic biomarkers for microtia (Figure 2F; Supplementary Table S7). Two overlapping genes were identified from the results of hub genes and SVM-RFE, namely, IL-6 and CXCL1 (Figure 2G), which may be specific biomarkers in microtia. Given the potential role of cytokines in microtia, IL-6, with the highest score in hub genes (Supplementary Table S6), has attracted our attention.

### Single-cell transcriptomic analysis of NECT and MECT

**FIGURE 3:**
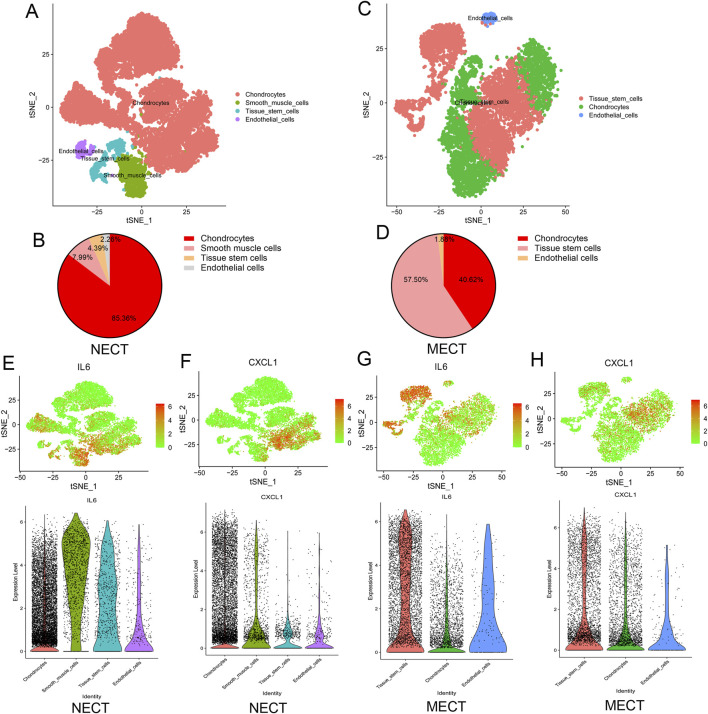
Single-cell transcriptomic analysis of NECT and MECT. **(A)** The cellular landscape of NECT by analysing Single Cell Transcriptomic datasets (GSE179135), the cell clusters were annotated according to various canonical based on expression of specific markers. **(B)** The proportion of different cells in NECT. **(C)** The cellular landscape of MECT by analysing Single Cell Transcriptomic datasets (GSE179135). **(D)** The proportion of different cells in MECT. **(E, F)** The expression of IL-6 and CXCL1 in different cells of NECT. **(G, H)** The expression of IL-6 and CXCL1 in different cells of MECT.

We further analyzed the single-cell transcriptomic data of microtia based on the GEO#GSE179135 dataset to explore the differences in the transcriptome and cellular landscape between NECT and MECT. As shown in Figures 3A,B, NECT was mainly composed of chondrocytes, with a proportion of 85.36%. However, in addition to chondrocytes (40.62%), a large number of tissue stem cells (57.50%) were present in MECT (Figures 3C,D). We then detected the expression of IL-6 and CXCL1 in NECT and MECT cells. In particular, IL-6 was mainly expressed in chondrocytes in NECT, while it was mainly expressed in tissue stem cells and had limited expression in chondrocytes in MECT (Figures 3E,G), whereas CXCL1 was primarily expressed in chondrocytes in NECT, while it was mainly concentrated in tissue stem cells and chondrocytes in MECT (Figures 3F,H).

### Deciphering the intercellular communication in microtia

**FIGURE 4:**
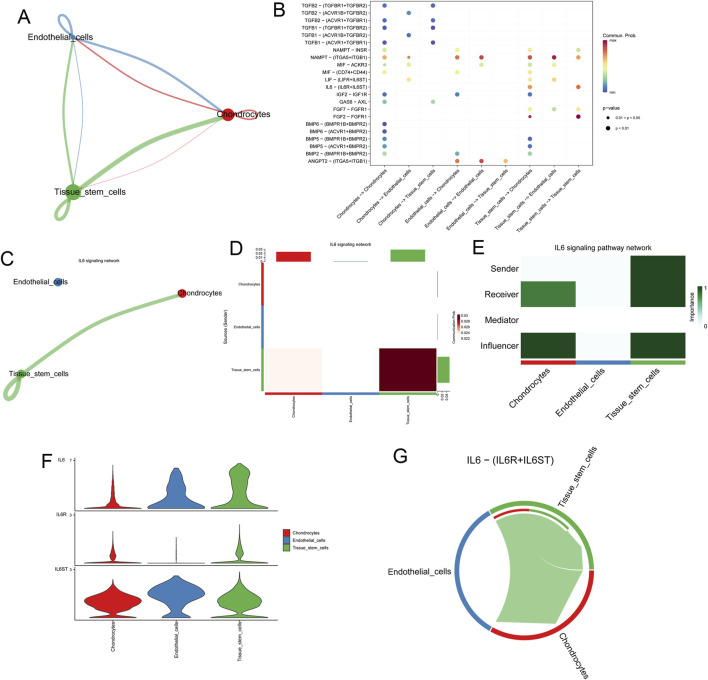
Deciphering the intercellular communication in microtia. **(A)** All the potential intercellular communication networks in microtia. **(B)** All possible intercellular pathways and ligand-receptor pairs in microtia. **(C, D)** Intercellular IL-6 signaling network involved by IL-6 in microtia. **(E)** In microtia, we analysed the roles of different cells in the IL-6 signaling pathway network, including sender, receiver, influencer, and mediator. **(F)** Expression of IL-6 and its receptor IL-6R and IL-6ST in different cells of microtia. **(G)** Intercellular communication involved in IL-6-(IL-6R + IL-6ST) signaling pathway in microtia.

Furthermore, we analyzed the co-expression of ligand, receptor, and target genes in MECT based on GEO#GSE179135 to detect the possible intercellular communication of microtia. Secreted signaling accounted for the largest proportion of intercellular communication (Supplementary Figure S1A). All potential intercellular communication networks were established in MECT, and communication between chondrocytes and tissue stem cells accounted for the largest weight (Figure 4A). The intercellular communication networks of different types of cells are shown in Supplementary Figures S1B–D. We detected all possible intercellular pathways and ligand-receptor pairs in MECT (Figure 4B) and found that IL-6 participates in the IL-6 signaling network between chondrocytes and tissue stem cells among the specific biomarkers of microtia (Figures 4C,D). Further analysis of the IL-6 signaling network revealed that tissue stem cells primarily act as senders to send ligands, tissue stem cells themselves, and chondrocytes act as the main receivers to receive signal molecules in MECT (Figure 4E). Moreover, IL-6 was mainly expressed in tissue stem cells, and its receptors, IL-6R and IL-6ST (GP130), were expressed in tissue stem cells and chondrocytes (Figure 4F). In addition, it should be noted that the IL6−IL6R + IL6ST pathway is mainly involved in the autocrine pathway of tissue stem cells and its interaction with chondrocytes in MECT (Figure 4G).

### IL-6 is downregulated in microtia

**FIGURE 5:**
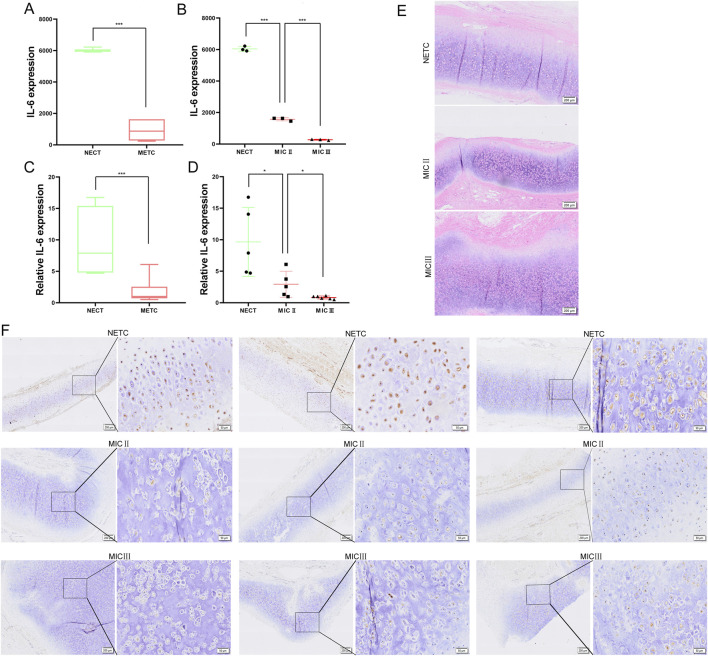
IL-6 is downregulated in microtia. **(A, B)** mRNA expression of IL-6 in three NECT and six MECT (including three MIC II tissues and three MIC III tissues) by analyzing transcriptome sequencing data. **(C, D)** mRNA expression of IL-6 in 5 NECT and 11 MECT (including 5 MIC II tissues and 6 MIC III tissues). **(E)** The HE staining of NECT and MECT images. Scale bar, 200 μm. **(F)** The protein level of IL-6 was detected in NECT (three different samples) and MECT (including three different MIC II tissues and three different MIC III tissues) using IHC, and we presented representative microscopic fields of the sample at different magnifications. Scale bars: 200 and 50 μm *P < 0.05, ***P < 0.001.

To further determine the role of IL-6 in microtia, we investigated the expression of IL-6 in three NECT and six MECT samples [including three ear cartilage tissues of microtia grade II (MIC II) and three ear cartilage tissues of microtia grade III (MIC III)] based on the transcriptome sequencing data of the samples we collected, and found that IL-6 was significantly downregulated in MECT (Figure 5A). Moreover, the expression of IL-6 was negatively associated with microtia severity (Figure 5B). To further verify the level of IL6 in microtia, we collected five NECT and 11 MECT (including five MIC II tissues and six MIC III tissues) to detect the mRNA expression of IL-6. We found that IL-6 expression was significantly decreased in MECT compared to NECT, and it decreased with an increase in the microtia grade (Figures 5C,D). Moreover, we also collected three NECT and six MECT samples (including three MIC II tissues and three MIC III tissues) to investigate the protein expression of IL-6. HE staining showed a disordered arrangement of chondrocytes in patients with microtia (Figure 5E). IHC showed that the protein level of IL-6 was also lower in MECT than in NECT (Figure 5F), and the negative control for IL-6 staining is shown in Supplementary Figure S1E.

### IL-6 signaling promotes the growth and migration of primary chondrocytes and PSCECs in microtia

**FIGURE 6:**
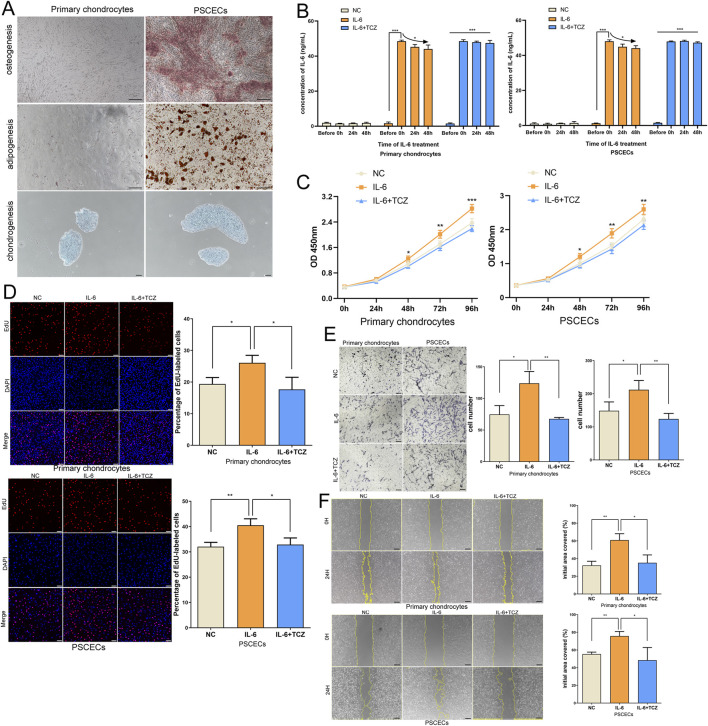
IL-6 signaling promotes the growth and migration of primary chondrocytes and PSCECs in microtia. **(A)** The ability of adipogenesis, osteogenesis, and chondrogenesis in PSCECs and primary chondrocytes after induction. Scale bar, 100 μm. **(B)** ELISA showed the change of IL-6 level in the cell culture medium after the addition of IL-6. **(C)** The proliferative ability of PSCECs and primary chondrocytes was determined by CCK8 assay in different groups. **(D)** The DNA synthesis of PSCECs and primary chondrocytes was detected by EdU assay, 50 μm. **(E)** The migrative capacity of primary chondrocytes and PSCECs treated by IL-6 or TCZ was assessed by transwell assay. Scale bar, 50 μm. **(F)** The migration of primary chondrocytes and PSCECs in different groups was detected by scratch wound assay. Scale bar, 100 μm *P < 0.05, **P < 0.01, ***P < 0.001.

To further verify the role of IL-6 in microtia, we extracted primary chondrocytes and PSCECs from ear cartilage tissues of the microtia. We found a significant difference in the morphology of primary chondrocytes and PSCECs (Supplementary Figure S2A). Flow cytometry showed that primary chondrocytes mainly expressed chondrocyte markers (COL2A1, SOX9, and ACAN), PSCECs mainly expressed stem cell markers (CD90, CD166, and CD44), and the expression rate of HLA-DR in PSCECs was very low (Supplementary Figures S2B,C). We found that PSCECs have a strong ability to induce adipogenesis, osteogenesis, and chondrogenesis, while primary chondrocytes have almost no osteogenic or adipogenic abilities, except for chondrogenesis (Figure 6A). Next, we added IL-6 to the culture medium of primary chondrocytes and PSCECs. Many studies have used concentrations of 50 or 100 ng/mL when treating cells with IL-6 (Rochfort and Cummins, 2015; Strickland et al., 2017), a order to provide sufficient IL-6 uptake to cells, we used a concentration of 50 ng/mL. ELISA showed a significant increase in IL-6 levels in the cell culture medium after the addition of IL-6, and there was a trend of consumption of IL-6 levels over time (Figure 6B). CCK-8 assays revealed that IL-6 significantly promoted the proliferation of primary chondrocytes and PSCECs (Figure 6C). EdU assay showed that the DNA replication activity of cells was significantly enhanced in the IL-6 treatment group (Figure 6D). Transwell assays showed that IL-6 promoted the migratory capacity of primary chondrocytes and PSCECs (Figure 6E). The scratch wound assays also demonstrated that the migration capacity of primary chondrocytes and PSCECs was strengthened by IL-6 (Figure 6F). In addition, tocilizumab (TCZ) competitively inhibits the binding of IL-6 and its receptors to block the IL-6 signaling pathway. We found that IL-6 levels in the culture medium did not change over time when added 50 ng/mL TCZ and 50 ng/mL IL-6 (Figure 6B). TCZ also reversed the effects of IL-6 on the proliferation, DNA replication, and migration of primary chondrocytes and PSCECs (Figures 6C–F).

### IL-6 binds to IL-6R and GP130 to activate the JAK/STAT3 and PI3K/AKT pathways in primary chondrocytes and PSCECs

**FIGURE 7:**
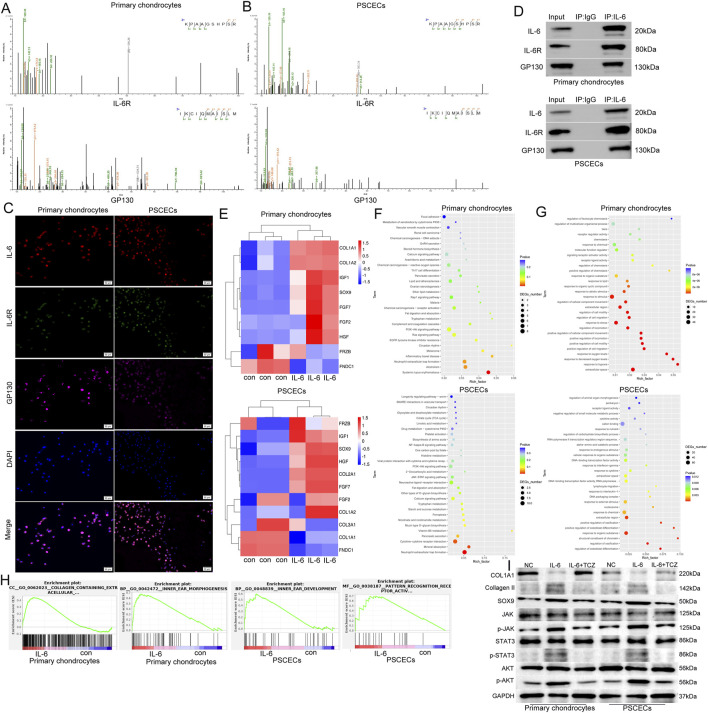
IL-6 binds to IL-6R and GP130 to activate the JAK/STAT3 and PI3K/AKT pathways in primary chondrocytes and PSCECs. **(A, B)** IP-MS identified receptors that bind to IL-6 among the membrane proteins of primary chondrocytes and PSCECs, and potential binding possibilities between IL-6, IL-6R, and GP130 were discovered. **(C)** IF assay showing the colocalization of IL-6, IL-6R, and GP130 in primary chondrocytes and PSCECs. Scale bar, 50 μm. **(D)** Co-IP confirmed the protein-binding relationship among IL-6, IL-6R, and GP130 in primary chondrocytes and PSCECs. **(E)** RNA-seq of primary chondrocytes and PSCECs detected the expression of cartilage fibrosis-related genes after IL-6 (50 ng/mL) stimulation. **(F)** Kyoto Encyclopedia of Genes and Genomes (KEGG) enrichment analysis of the upregulated genes in primary chondrocytes and PSCECs after IL-6 stimulation showcased the signaling pathways involved in these genes. **(G)** GO enrichment analysis of the upregulated genes in primary chondrocytes and PSCECs after IL-6 stimulation. **(H)** GSEA revealed that the gene terms for extracellular collagen, ear morphogenesis, pattern cognitive receptor activation, and ear development were highly enriched in the IL-6 treatment group of primary chondrocytes and PSCECs. **(I)** Western blots showing the protein levels of SOX9, COL1A1, Collagen II and JAK/STAT3 and PI3K/AKT signaling pathways in different groups; GAPDH was used as a control.

To explore the mechanism of IL-6, we used immunoprecipitation to obtain protein complexes that interact with IL-6, and then identified the protein complexes using MS. We found that IL-6 binds to IL-6R and GP130 among membrane proteins of primary chondrocytes and PSCECs (Figures 7A,B). The IF assay showed that IL-6, IL-6R, and GP130 were co-located in the area outside the nucleus (Figure 7C). Co-IP further confirmed the binding between IL-6 and its receptor proteins, IL-6R and GP130, in primary chondrocytes and PSCECs (Figure 7D). To identify the activation status of the downstream pathways of IL-6 signaling, we conducted RNA-seq of primary chondrocytes and PSCECs after IL-6 (50 ng/mL) stimulation. In primary chondrocytes, the expression of most cartilage protection genes (SOX9, FGF2, IGF1, and HGF) increased, while the expression of cartilage fibrosis-related gene (FNDC1) decreased in the IL-6 treatment group. In PSCECs (Figure 7E), we found that many cartilage protection genes (COL2A1, SOX9, IGF1, and HGF) were upregulated, whereas cartilage fibrosis-related genes (COL1A1, COL3A1, and FNDC1) were downregulated after IL-6 stimulation (Figure 7E). KEGG analysis showed that the upregulated genes in primary chondrocytes were enriched in the PI3K−AKT pathway, calcium signaling pathway, and receptor activation, while the upregulated genes in PSCECs were enriched in cytokine-cytokine receptor interaction, JAK-STAT signaling pathway, and PI3K-AKT signaling pathway (Figure 7F). This suggests that the JAK/STAT3 and PI3K/AKT pathways may be key downstream pathways of IL-6 signaling. GO analysis indicated that the upregulated genes in primary chondrocytes were involved in positive regulation of cell migration, positive regulation of cell motility, and response to stimulus, and the upregulated genes in PSCECs were involved in response to cytokines, DNA-binding transcription factor activity, and cell differentiation (Figure 7G). Moreover, Gene Set Enrichment Analysis (GSEA) revealed that the gene terms of extracellular collagen and ear morphogenesis were highly enriched in the IL-6 treatment group of primary chondrocytes, and the gene set of pattern cognitive receptor activation and ear development was highly enriched in the IL-6 treatment group of PSCECs (Figure 7H). Moreover, we found that IL-6 promoted the expression of cartilage development-related proteins (SOX9 and Collagen II), while inhibiting the expression of cartilage fibrosis protein (COL1A1) in primary chondrocytes and PSCECs (Figure 7I). We also demonstrated that the JAK/STAT3 and PI3K/AKT pathways were activated after IL-6 stimulation, and that TCZ reversed the effect of IL-6 on the expression of related proteins (Figure 7I). These results indicate that IL-6 is closely related to the motility and differentiation of cells in the ear cartilage tissue and the formation and development of ear tissue.

### IL-6 signaling participates in the intercellular communication network between PSCECs and primary chondrocytes

**FIGURE 8:**
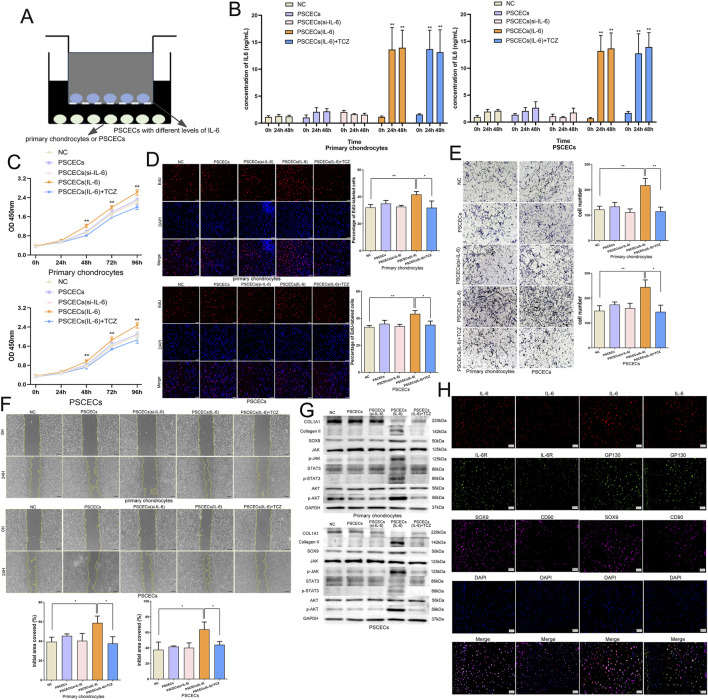
IL-6 signaling participates in the intercellular communication network between PSCECs and primary chondrocytes. **(A)** The cell co-culture system using the transwell plate with a pore size of 0.4 μm. **(B)** ELISA showed the change of IL-6 level in the cell culture medium after co-cultured with PSCECs. **(C)** The proliferative ability of PSCECs and primary chondrocytes was determined by CCK8 assay in different groups. **(D)** The DNA synthesis of PSCECs and primary chondrocytes was detected by EdU assay. Scale bar, 50 μm. **(E)** The migrative capacity of primary chondrocytes and PSCECs in different groups was assessed by transwell assay. Scale bar, 50 μm. **(F)** The migration of primary chondrocytes and PSCECs in different groups was detected by scratch wound assay. Scale bar, 100 μm. **(G)** Western blots identified the protein level of SOX9, COL1A1, Collagen II and JAK/STAT3 and PI3K/AKT components pathway in different groups, GAPDH was used as a control. **(H)** In microtia, mIHC assay showed that IL-6, IL-6R and GP130 co-expressed in CD90^+^ stem cells and SOX9+ chondrocytes. Scale bar, 50 μm *P < 0.05, **P < 0.01.

To explore the influence of IL-6 on the intercellular communication network between PSCECs and primary chondrocytes, PSCECs overexpressing and low-expressing IL6 were constructed using IL-6 overexpression lentivirus and the siRNA of IL-6 (Supplementary Figure S3A). We found a significant increase in IL-6 levels in the culture medium of the overexpressing IL-6 PSCECs (Supplementary Figure S3B). Next, we constructed a cell co-culture system using a transwell plate with a pore size of 0.4 um to investigate the effects of PSCECs with different levels of IL-6 on themselves and primary chondrocytes (Figure 8A). ELISA results showed that IL-6 levels in the culture medium were significantly increased in both PSCECs and primary chondrocytes after co-culture with PSCECs overexpressing IL-6 (Figure 8B). The proliferative ability of primary chondrocytes and PSCECs was significantly increased after co-culture with PSCECs secreting high levels of IL-6 (Figure 8C). EdU assay also showed that the DNA replication activity of primary cells was promoted after treatment with PSCECs overexpressing IL-6 (Figure 8D). Transwell and scratch wound assays demonstrated that the migratory capacity of primary chondrocytes and PSCECs was strengthened by co-culture with PSCECs secreting high levels of IL-6 (Figures 8E,F). TCZ also abolished the effect of IL-6 secreted by PSCECs on the proliferation, DNA replication, and migration of primary cells (Figures 8C–F). Meanwhile, we found that the expression of cartilage development-related proteins (SOX9 and Collagen II) in primary cells was upregulated and the JAK/STAT3 and PI3K/AKT pathways were activated after co-culture with PSCECs secreting high levels of IL-6, while the expression of cartilage fibrosis protein (COL1A1) was downregulated (Figure 8G). TCZ abolished the effect of IL-6 secreted by PSCECs on the expression of the related proteins (Figure 8G). Furthermore, mIHC was used to confirm intercellular communication in MECT. IL-6, IL-6R, and GP130 were co-expressed in CD90^+^ stem cells and SOX9+ chondrocytes in the microtia (Figure 8H).

### IL-6 promotes the formation of cartilage microspheres

**FIGURE 9:**
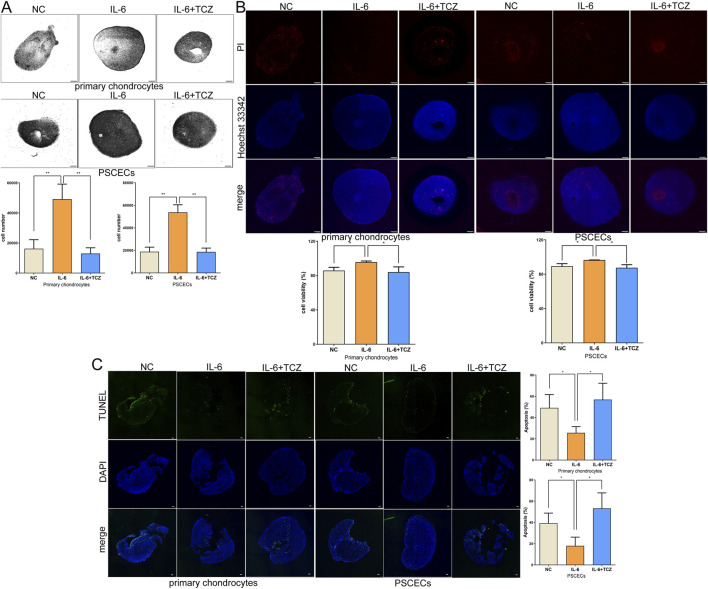
IL-6 promotes the formation of cartilage microspheres. **(A)** The number of total cells in the cartilage microspheres formed by PSCECs and primary chondrocytes. Scale bar, 500 μm. **(B)** The cartilage microspheres were stained by using Hoechst 33,342 and Propidium Iodide and the cell viability were calculated. Scale bar, 500 μm. **(C)** TUNEL assays measured the number of apoptotic cells in cartilage microspheres. Scale bar, 100 μm *P < 0.05, **P < 0.01.

To further investigate the role of IL-6 in ear cartilage regeneration, we conducted a 3D-culture of PSCECs and primary chondrocytes to construct cartilage microspheres. We added IL-6 and TCZ to the growth environment of primary chondrocytes and PSCECs at a concentration of 50 ng/mL. As shown in Figure 9A, the total number of cells in the cartilage microspheres formed by PSCECs and primary chondrocytes was significantly increased in the IL-6 treatment group. We calculated cell viability in cartilage microspheres and found a higher proportion of live cells after IL-6 stimulation (Figure 9B). The TUNEL assay showed that IL-6 significantly inhibited the apoptosis of primary cells in the cartilage microspheres (Figure 9C). We also found that TCZ reversed the effect of IL-6 on cell number, viability, and apoptosis in cartilage microspheres (Figures 9A–C).

### IL-6 promotes the development of embryonic ear *in vivo*

**FIGURE 10:**
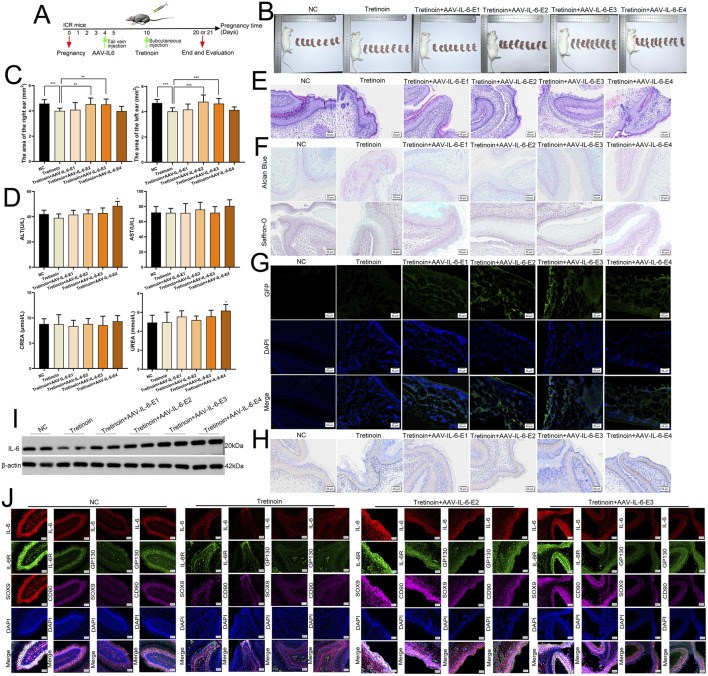
IL-6 promotes the development of embryonic ear *in vivo*. **(A)** We used tretinoin to construct the animal model of microtia, and we used AAV-IL-6 for gene therapy. **(B)** Representative images of pregnant mice and their fetuses after birth. **(C)** The area of the fetal mice ear in different group. **(D)** The liver and kidney function in pregnant mice. **(E)** The HE staining of fetal mice ear. Scale bar, 50 μm. **(F)** The Alcian Blue and Saffron-O staining of fetal mice ear. Scale bar, 50 μm. **(G)** The frozen section was used to detect GFP in fetal mice ear. Scale bar, 50 μm. **(H)** The protein level of IL-6 was detected in fetal mice ear by using IHC. Scale bar, 50 and 20 μm. **(I)** The protein level of IL-6 was detected in fetal mice ear by using Western blots, β-was used as a control. **(J)** mIHC assay showed that IL-6, IL-6R and GP130 co-expressed in CD90^+^ cells and SOX9+ cells in the ears of fetal mice. Scale bar, 50 μm *P < 0.05, **P < 0.01.

Previous studies have not reported the impact on ear development in mice after knocking down IL-6. Embryonic development is closely related not only to oneself but also to the mother’s condition. To confirm the role of IL-6 in microtia *in vivo*, we constructed a mouse model of microtia by subcutaneous injection of tretinoin (a drug with a clear teratogenic effect, especially craniofacial deformities) into pregnant mice on the 10th day of pregnancy (Figure 10A). We performed gene therapy in pregnant mice on the fourth day of pregnancy using AAV-IL-6 at escalating doses to prevent microtia (Figure 10A), and treatment groups were designated as AAV-IL-6-E1 (low dose, 5 × 10^10^ GC), AAV-IL-6-E2 (medium-low dose, 1 × 10^11^ GC), AAV-IL-6-E3 (medium-high dose, 3 × 10^11^ GC), and AAV-IL-6-E4 (high dose, 5 × 10^11^ GC), respectively. Representative images of the pregnant mice and their fetuses after birth are shown in Figure 10B. We measured the area of the fetal mouse ear and found that tretinoin can inhibit the development of the fetal ear (Figure 10C). Medium dose of AAV-IL-6 (1 × 10^11^ GC and 3 × 10^11^ GC) reversed the underdevelopment of the ear caused by tretinoin (Figure 10C). Low and medium dose of AAV-IL-6 and tretinoin did not adversely affect liver and kidney function in pregnant mice, but high-dose of AAV-IL6 had some adverse effects on it (Figure 10D). HE staining showed a disordered arrangement of chondrocytes in the tretinoin-treated group and an orderly arrangement in the control and medium dose of AAV-IL-6 treatment group (Figure 10E). We found that the staining of Alcian Blue and Saffron-O in the tretinoin group was light, indicating less cartilage matrix, whereas the medium dose of AAV-IL-6 treatment group had more cartilage matrix (Figure 10F). Frozen sections showed that AAV could carry IL-6 through the placental barrier and transport it to the ears of fetal mice (Figure 10G). Meanwhile, the protein level of IL-6 was found to be relatively low in the tretinoin group, and gradually increased with the increase of AAV-IL-6 treatment dose (Figures 10H,I). Furthermore, mIHC confirmed that IL-6, IL-6R, and GP130 were co-expressed in CD90^+^ cells and SOX9+ cells in the ears of fetal mice, indicating the intercellular communication involved by IL-6 in the development of the ear *in vivo* (Figure 10I). These results confirmed the potential role of IL-6 in gene therapy for microtia.

## Discussion

Among congenital craniofacial deformities, the incidence of microtia was the second highest (Guo et al., 2021). Microtia not only affects children’s appearance but also causes psychological and social behavioral problems due to ridicule or alienation from peers (Ramprasad et al., 2020; Zhou et al., 2022). Auricular reconstruction is the main treatment method for microtia, but it requires the rib cartilage of the patient as a scaffold, which can lead to pain, scars, and clicking sensation of the chest and chest contour deformity in the patient (Uppal et al., 2008). Therefore, it is necessary to study the pathogenesis of microtia. Many scholars have conducted extensive research on the etiology of microtia. Zhang et al. found that SOX8 may be related to dysregulation of cartilage development, whereas EGR1 may be related to dysregulation of the oxidative stress response in microtia (Ma et al., 2022). Scholars have also found that mutations located between ROBO1 and ROBO2 increase the risk of microtia in American indigenous populations (Quiat et al., 2022). However, to date, no clear pathogenic genes have been identified in the microtia. Given that patients with microtia exhibit underdevelopment of auricular cartilage tissue, it is crucial to explore the key genes and signaling pathways involved in the development of auricular cartilage.

In this study, we first identified 1,077 differentially expressed genes in microtia using transcriptome sequencing data from the samples we collected. We conducted WGCNA to search for genes with significant clinical relevance to microtia and found 2,532 microtia-associated genes. We identified 621 key genes by considering the intersection of these genes. A PPI network based on 621 key genes was built, and 15 hub genes of the network were identified. Moreover, we screened 37 genes as potential diagnostic biomarkers for microtia through SVM-RFE and intersected them with 15 hub genes to obtain two specific biomarkers (IL-6 and CXCL1) in microtia. GO and KEGG enrichment analyses suggested that cytokine and intercellular communication may play important roles in microtia. Intercellular communication mediated by cytokine receptors is closely related to various biological processes, such as cell differentiation and organ development (Huang et al., 2021; Tsang et al., 2017). For deepening our understanding of the pathogenesis of microtia, we found that normal ear cartilage tissue is mainly contain chondrocytes, while microtia ear cartilage tissue is mainly composed of chondrocytes and tissue stem cells through analyzing single-cell transcriptome data. We further found that IL-6 signaling is the main intercellular communication pathway involved in the autocrine function of tissue stem cells and their interaction with chondrocytes.

IL-6 is a cytokine with both proinflammatory and anti-inflammatory functions (Scheller et al., 2011). IL-6 not only participates in the development of arthritis, autoimmune diseases, and cancer (Hong et al., 2022; Johnson et al., 2018; Tanaka et al., 2014), but also plays a role in tissue repair. It has been proven that IL-6 can maintain joint cartilage regeneration and promote corneal repair of corneal (Arranz-Valsero et al., 2014; Liu et al., 2022). However, the role of IL-6 in the microtia has not yet been studied. Here, we found that IL-6 was significantly underexpressed in the ear cartilage tissue of the microtia. To further verify the role of IL-6 in microtia, we extracted primary chondrocytes and PSCECs from cartilage tissues of microtia and confirmed the cells by detecting cell molecular markers and inducing multidirectional differentiation of cells. IL-6 has been shown to promote the growth, DNA replication activity, and migration ability of chondrocytes and PSCECs, and TCZ can reverse the effect of IL-6. Next, we explored the mechanism of IL-6 in microtia and found that IL-6 binds to IL-6R and GP130 in primary chondrocytes and PSCECs. IF and co-IP further confirmed the binding between IL-6 and its receptors IL-6R and GP130. RNA-seq of primary chondrocytes and PSCECs showed that the expression of most cartilage protection genes was increased after IL-6 stimulation, and the JAK/STAT3 and PI3K/AKT pathways may be the key downstream pathways of IL-6. We further confirmed the activation of JAK/STAT3 and PI3K/AKT pathways. GSEA also showed that IL-6 treatment is closely related to ear morphogenesis and development. These results indicate that IL-6 is crucial for the movement and differentiation of cells in ear cartilage tissue.

Three signaling pathways are involved in IL-6 (Choudhary et al., 2016; Heink et al., 2017; Hunter and Jones, 2015; Kang and Kishimoto, 2021): classic signaling, trans-signaling, and cluster presentation. In classical signaling, IL-6 binds to its receptor IL-6R and then binds to GP130 to initiate intracellular signaling, Trans-signaling occurs in cells that do not express IL-6R. IL-6R exists in a soluble form (sIL-6R), IL-6 binds to sIL-6R, and then binds to GP130. In cluster presentation, IL-6 binds to IL-6R within DC cells and is then transported to the cell membrane for binding to GP130. Here, we found that IL-6R is expressed in the ear cartilage tissue of the microtia. Moreover, IL-6R is expressed in primary chondrocytes and PSCECs. These results indicated that IL-6 activates downstream pathways through classical signaling pathways. Moreover, we confirmed that IL-6 signaling and its downstream JAK/STAT3 and PI3K/AKT pathways are involved in the autocrine of PSCECs and communication between PSCECs and chondrocytes by constructing a cell co-culture system. In addition, we simulated the cell growth environment *in vivo* and cultivated cartilage microspheres to demonstrate the role of IL-6 in ear cartilage regeneration. Finally, we constructed a mouse model of congenital microtia and used an IL-6 overexpression vector constructed using AAV to conduct preventive intervention in pregnant mice, and found that IL-6 is a potential therapeutic target for microtia. However, this study had some limitations. The use of IL-6 for the clinical treatment or prevention of microtia has not been specifically discussed in this article, especially regarding the selection of its therapeutic concentration. This is an extremely complex issue that deserves further research.

In summary, we identified key genes and signaling pathways in microtia using bioinformatics analysis. IL-6 signaling was found to play an important role in the development and regeneration of auricular cartilage in the microtia. IL-6 signaling and its downstream pathways participate in communication between PSCECs and chondrocytes. IL-6 is a potential biomarker and preventive and therapeutic target for microtia.

## Methods

### Data acquisition, human tissue samples and RNA-seq

We searched the term “microtia” and “single cell” in The Gene Expression Omnibus (GEO) website (https://www.ncbi.nlm.nih.gov/geo/). The GEO#GSE179135 dataset, which included six normal ear cartilage tissues and three microtia ear cartilage tissues, was used for single-cell and intercellular communication analysis. Eleven normal ear cartilage tissues and 23 microtia ear cartilage tissues were collected from patients at the Plastic Surgery Hospital of the Chinese Academy of Medical Sciences and Peking Union Medical College. Samples were stored in liquid nitrogen or fixed with 4% paraformaldehyde after collection according to different experimental requirements. Three normal ear cartilage tissues and six microtia ear cartilage tissues (three MIC II and three MIC III tissues) were used for RNA-seq. The RNA-seq results were deposited in GEO#GSE227119. This study was approved by the Ethics Committee of the Plastic Surgery Hospital of the Chinese Academy of Medical Sciences and Peking Union Medical College, and informed consent was obtained from all participants.

### Bioinformatics analysis

We analyzed the genomic profiles of normal ear cartilage tissue and microtia ear cartilage tissue to identify differentially expressed genes (DEGs). The NormalizeBetweenArrays algorithm, heatmap, and ggplot2 packages in R were used. For the WGCNA analysis, the WGCNA package in R software was used, and we calculated and identified modules with significant clinical relevance. We used ggplot2, org.Hs.eg.db, enrichplot, circlize, RColorBrewer, ggpubr, dplyr, complexHeatmap, and clusterProfiler in the R project to conduct GO and KEGG enrichment analyses. The protein-protein interaction relationships were obtained using the STRING website (https://cn.string-db.org/) and Cytoscape software, and hub genes were identified using the cytoHubba plugin of Cytoscape. Support Vector Machine-recursive feature elimination (SVM-RFE) was used to identify diagnostic biomarkers with superior discriminative ability in microtia, using the e1071, kernlab, and caret packages. We compared the cellular landscape and transcriptome between normal ear cartilage tissue and microtia ear cartilage tissue using the GEO#GSE179135 dataset. The SingleR package was used to conduct single-cell transcriptomic analysis. The Sqjin/CellChat package was used to conduct intercellular communication analyses. A detailed explanation of the bioinformatic analysis methods is provided in Supplementary Material S1.

### Extraction of primary cells lines, cell culture, biological reagents, oligonucleotides and transfection

The ear cartilage tissue was cut into 1 mm^3^ fragments and digested with 0.25% trypsin (Gibco, United States) for 30 min. Next, collagenase IV (Biotopped, Beijing, China) was added for overnight digestion, filtered through a 200-mesh cell sieve, and centrifuged to obtain cell precipitates. The cells were inoculated into culture bottles to obtain primary chondrocytes. For extraction of PSCECs, ear cartilage tissues were cut into 1 mm^3^ fragments, and 0.35% collagenase P (Roche, Germany) and 0.2% dispase (Sigma-Aldrich, United States) were added to cartilage tissues for digestion 1.5 h followed by filtering through a 100-mesh cell sieve. The cells were inoculated into a culture dish pre-coated with laminin (Sigma-Aldrich, United States) at a density of 600 cells/mL. The medium was changed to remove the non-adherent cells after 30 min. When a single cell formed a colony, the colony was digested and picked out using a cloning ring, and then inoculated into a new culture bottle to obtain PSCECs. Chondrocytes were grown in Dulbecco’s modified Eagle’s medium (DMEM)/High Glucose (Cytiva, United States) supplemented with 10% fetal bovine serum (Pricella, Wuhan, China). PSCECs were maintained in complete medium for mesenchymal stem cells (Oricell, Guangzhou, China). All cell lines were placed in a humidified cell incubator with 5% CO_2_ at 37°C. Cytokine IL-6 was purchased from PeproTech (New Jersey, United States) and tocilizumab was obtained from MedChemExpress (New Jersey, United States). The small interfering RNA (siRNA) for IL-6 was chemically synthesized by Hippo Biology (Huzhou, China), and the sequence was as follows: 5′-GCAGGACAUGACAACUCAUTT-3′. Lipofectamine 3000 (Invitrogen, United States) was used to transfect the oligonucleotides. The IL-6 overexpression lentivirus vector was constructed by Zebrafish Biotechnology Co., Ltd. (Nanjing, China), and stably overexpressing IL-6 cells were built by infecting cells with the lentivirus.

### Adipogenesis, osteogenesis, and chondrogenesis assay of primary cells

A mesenchymal stem cell lipid-induced differentiation kit (Oricell, Guangzhou, China) was used for adipogenesis. Primary cells were seeded in a six-well plate pre-coated with gelatin. We then added adipogenic induction differentiation media A and B for alternating cultivation. The cells were fixed with polyformaldehyde and lipid droplets were observed using oil red staining after 3 weeks. A mesenchymal stem cell osteogenic-induced differentiation kit (Oricell, Guangzhou, China) was used for osteogenesis. The cells were seeded in a six-well plate pre-coated with gelatin and cultured in osteogenic induction differentiation medium. After 3 weeks, the cells were fixed, and calcium deposition was observed using alizarin red staining. A mesenchymal stem cell chondrogenic-induced differentiation kit (Oricell, Guangzhou, China) was used for chondrogenesis. Primary cells were transferred into 15 mL centrifuge tubes and cultured in chondrogenic induction differentiation medium to form cartilage microspheres. After 3 weeks, the microspheres were fixed and prepared into paraffin sections, and acidic mucopolysaccharides were observed using alisin blue staining.

### Flow cytometry

Flow cytometry was used to detect the expression of chondrocyte and stem cell markers in the primary cells. In PSCECs, directly labeled antibodies against CD90, CD166, CD44, and HLA-DR were obtained from BioLegend (California, United States). Primary chondrocytes were incubated with antibodies against SOX9 (Proteintech, Chicago, United States), ACAN (Proteintech, Chicago, United States), and COL2A1 (ProMab, Hunan, China), followed by incubation with goat anti-mouse IgG HL (ALEXA Fluor 488) (Abcam, Cambridge, UK). Isotype control antibodies were obtained from BioLegend (California, United States).

### Enzyme linked immunosorbent assay (ELISA)

To detect the IL-6 content in the medium, a human IL-6 Quick ELISA Kit (BOSTER, Wuhan, China) was used. The sample and standard were added to a 96-well plate pre-coated with anti-human IL-6 antibody, and HPR linked antibody were added for oscillatory incubation at room temperature for 60 min, We added TMB color developing reagent after washing with TBST-T, and mixed in the stop solution 20 min later. Absorbance was measured at 450 nm using a microplate reader. We constructed a standard curve and calculated the IL-6 content in the samples.

### Cell proliferation assay

For the cell counting kit-8 (CCK-8, Beyotime, Shanghai, China) assay, the corresponding cells onto a 96 well plate (3,000 cells per well). At different time points (0, 24, 48, 72, and 96 h), CCK8 solution was added to each well and incubated for an additional 2 h. A microplate reader was used to detect the absorbance at an optical density of 450 nm. For the EdU assay, the DNA replication activity of primary cells was evaluated using an EdU Cell Proliferation Kit with Alexa Fluor 555 (Beyotime, Shanghai, China). Experiments were conducted according to the manufacturer’s instructions. Cells were visualized using a Leica laser scanning confocal microscope and photographed.

### Cell migration assays

For the transwell assay, the corresponding primary cells were resuspended in serum-free culture medium and placed in a small chamber of a transwell culture plate (Croning, United States), with a pore size of 8.0 μm. A culture medium containing 10% fetal bovine serum was added to the lower chamber. Migrating cells were fixed and stained with crystal violet for 24 h. Finally, photos were taken, and cells were counted. For the scratch wound assay, the primary cells were inoculated into six-well plates, and then the cells were scratched using a 200 μL pipette to create a wound space. Wound widths were recorded at 0 and 24 h.

### Extraction of RNA and quantitative RT-PCR (qRT-PCR)

Total RNA was extracted from cells and tissues using a Total RNA Extraction Kit (Solarbio, Beijing, China) according to the manufacturer’s instructions. Reverse transcription (RT) was performed using HiScript® II Q RT SuperMix for qPCR (Vazyme Biotech, Nanjing, China). The AceQ qPCR SYBR Green Master Mix (Vazyme Biotech, Nanjing, China) was used for qRT-PCR. The Quant Gene 9600 Real-time Fluorescence Quantitative PCR Detection System (Bioer, Hangzhou, China) was used for amplification according to predetermined reaction conditions. GAPDH was used for normalization. The primers for IL-6 and GAPDH were synthesized using the following sequences: IL-6, 5′-AAAGAGGCACTGGCAGAAAA-3′ (forward) and 5′-TTTCACCAGGCAAGTCTCCT-3′ (reverse); GAPDH, 5′-ACCCAGAAGACTGTGGATGG-3′ (forward) and 5′-TTCAGCTCAGGGATGACCTT-3′ (reverse). Data were calculated using 2^–△△Ct^.

### Western blot analysis

Total protein was extracted from cells using a Total Protein Extraction Kit (KenGen BioTech, Nanjing, China) and quantitatively detected using the bicinchoninic acid Protein Assay Kit (KenGen BioTech, Nanjing, China). We added the equal amounts of protein in to the SDS-PAGE, and the protein were separated according to the conditions and transferred to polyvinylidene fluoride membranes (Millipore, Billerica, United States). The membranes were blocked and incubated overnight with diluted antibodies against COL1A1 (1:1,000, Cell Signaling Technology, United States), Collagen II (1:1,000, Abcam, UK), SOX9 (1:5,000, Proteintech, United States), JAK (1:1,000, Affinity Biosciences, United States), phospho-JAK (1:1,000, Affinity Biosciences, UK), STAT3 (1:1,000, Affinity Biosciences, UK), phospho-STAT3 (1:1,000, Affinity Biosciences, UK), AKT (1:5,000, Proteintech, United States), and phospho-AKT (1:2,000, Affinity Biosciences, UK). Membranes were incubated with HRP-conjugated secondary antibody (ABclonal, Wuhan, Chian), and we visualized the results through exposure. GAPDH and β-actin was used for normalization (1:50,000, Proteintech).

### Immunoprecipitation mass spectrometry (IP-MS)

Immunoprecipitation was used to enrich and obtain protein complexes, and an antibody against IL-6 (Cell Signaling Technology, CST, United States) was used for immunoprecipitation. After reduction and alkylation treatment, the protein complex was enzymatically hydrolyzed at 37°C for 20 h. After desalination, the enzymatic hydrolysis products were freeze-dried and re-dissolved in 0.1% FA solution. The sample was separated by chromatography and analyzed using a Q Exactive-HFX mass spectrometer (Thermo Scientific, United States).

### Immunofluorescence (IF) and multiplex immunohistochemical (mIHC)

For IF, we prepared cell smears, fixed cells with paraformaldehyde, and used 0.5% TritonX100 for cell permeability. Next, we used 3% bovine serum albumin for blocking 1 h and incubated the slices with primary antibody at 4°C overnight. Antibodies against IL-6 (1:300; Proteintech, Chicago, IL, United States), IL-6R (Abcam, 1:1,000, Cambridge, UK), and GP130 (Proteintech, 1:1,000, Chicago, IL, United States) were used. After incubation with the secondary antibody (ZSGB-BIO, Beijing, China) at room temperature for 1 h, we used to stain the cell nucleus and the cells were photographed under a fluorescence microscope. For mIHC, we dewaxed the slices and used a graded alcohol series for hydration. Antigen repair was conducted by boiling it in the repair solution at 95°C for 15–20 min, and 10% BSA was used for blocking. Primary antibodies against IL-6 (1:300, Proteintech, Chicago, United States), IL-6R (Abcam, 1:1,000, Cambridge, UK), GP130 (Proteintech, 1:1,000, Chicago, United States), CD90 (Abcam, 1:600, Cambridgeshire, UK), and SOX9 (Abcam, 1:1,000, Cambridge, UK) were used to incubate the slices at 4°C overnight. We added TSA fluorescent dye reaction solution onto the slice after incubation with the secondary antibody (ZSGB-BIO, Beijing, China), repeated the steps after antigen repair, and continued to label other proteins. DAPI was used to stain the cell nuclei. We observed and photographed the slices under an OLYMPUS microscope.

### Co-immunoprecipitation (Co-IP)

We collected cells, added cell lysis buffer and protease inhibitor, and centrifuged it to collect the supernatant for later use. Next, 400 μL of a combining/washing buffer solution (PBST) was added to 40 μL of Protein A/G Magnetic Beads (MedChemExpress, New Jersey, United States) and placed on a magnetic rack for magnetic separation. Antibodies against IL-6 (40 μg/mL, Cell Signaling Technology, CST, United States) were added to the magnetic beads, and sufficient suspension and incubation were performed. Magnetic beads were collected and washed repeatedly. Prepared antigen samples (400 μL) were added for thorough suspension and incubation. We added 50 μL 1×SDS-PAGE Loading Buffer into the magnetic beads at 95°C for 5 min to separate the magnetic beads. The supernatant was collected for SDS-PAGE detection, and antibodies against IL-6R (Abcam, Cambridge, UK) and GP130 (Proteintech, Chicago, United States) were used for subsequent analysis. We used an optimized secondary antibody that recognizes only the primary antibodies in their natural state (Abcam, Cambridge, UK).

### RNA-sequencing (RNA-Seq)

We added IL-6 to the culture medium at a concentration of 50 ng/mL, and extracted total RNA from primary chondrocytes and PSCECs of three control groups and three IL-6 treatment groups after 48 h. We commissioned BIOZERON (Shanghai, China) for RNA-seq analysis. The Illumina TruseqTM RNA Sample Pre-Kit method was used to construct the library, and the basic principle of sequencing was Sequencing by Synthesis. We analyzed the mRNA sequence and abundance information to identify changes in the expression of downstream pathways at the transcriptional level in primary cells after IL-6 stimulation.

### Establishment of cell co-culture system

We chose a Transwell chamber with a pore size of 0.4 um to construct a cell co-culture system, which only allows cytokines to pass through. We inoculated PSCECs with different levels of IL-6 into the transwell chamber and cultured the cells continuously until they adhered to the membrane. The untreated primary chondrocytes or PSCECs were seeded into transwell culture plates and cultured until the cells adhered to the wall. We placed the transwell chamber into the culture plate and added new culture medium for 48 h of co-culture. The cells in the culture plate were subjected to subsequent experiments after coculture.

### Culture of cartilage microspheres and calculation of cell viability

A mesenchymal stem cell chondrogenic-induced differentiation kit (Oricell, Guangzhou, China) was used to 3D-culture of primary cells. The cells were transferred into 15 mL centrifuge tubes and cultured in chondrogenic induction differentiation medium. The medium was changed every 3 days, and the cell colonies were suspended in the culture medium to form cartilage microspheres. After 3 weeks, the cartilage microspheres were stained with Hoechst 33342 (Invitrogen, United States) and Propidium Iodide (PI) (Invitrogen, United States). The cells were visualized using a Leica laser scanning confocal microscope and cell viability was calculated.

### TUNEL assay

We used the One Step TUNEL Apoptosis Assay Kit (Beyotime, Shanghai, China) to detect apoptosis in the cartilage microspheres. The cartilage microspheres were fixed using paraformaldehyde, embedded in paraffin, and sliced into sections. The sections were dewaxed and rehydrated and protease K was added without DNase for 30 min of incubation. The TUNEL detection solution was added to the sample and incubated in the dark for 60 min. We used DAPI to stain the cell nucleus and photographed under a Leica laser scanning confocal microscope.

### Construction of the mice model of microtia

Sixteen pregnant ICR mice were purchased from Biocytogen (Beijing, China). Four mice were excluded from the experiment due to false pregnancies, and 12 pregnant mice were ultimately included in the experiment. To construct the fetal mouse model with microtia, 30 mg/kg tretinoin (Merck, Germany), a drug with teratogenic effects (especially craniofacial deformities), was subcutaneously injected into pregnant mice on the 10th day of pregnancy. The IL-6 overexpression vector constructed using AAV was synthesized by MIJIA (Beijing, China). Escalating doses (5 × 10^10^ GC, 1 × 10^11^ GC, 3 × 10^11^ GC and 5 × 10^11^ GC) AAV-IL-6 was injected into pregnant mice via the tail vein on the fourth day of pregnancy to perform gene therapy. On the 20th or 21st day of pregnancy, we measured the area of the area of newborn mice, as well as liver and kidney functions (ALT, AST, UREA, and CREA) in pregnant mice. This study was approved by the Ethics Committee of the Plastic Surgery Hospital of the Chinese Academy of Medical Sciences and the Peking Union Medical College.

### Frozen sections, hematoxylin-eosin (HE), Alcian Blue, and Saffron-O staining and immunohistochemistry (IHC) staining

For frozen section, fresh fetal mouse ear tissue was immediately frozen at −80°C, and were embedded with frozen embedding agent, and sliced using a constant cold box slicer. We observed and photographed the slices under an OLYMPUS microscope. For HE staining, sections were incubated with hematoxylin after being dewaxed with xylene, rehydrated with graded alcohol, and then stained with eosin. Next, the sections were dehydrated in a graded alcohol series and clarified in xylene. Representative images were taken using a microscope after sealing the sections with neutral resin. For Alcian Blue and Saffron-O staining, the experimental steps were almost identical to those for HE staining. Alcian Blue was purchased from Beyotime (Shanghai, China), and Saffron-O was purchased from Solarbio (Beijing, China). For IHC staining, sections were dewaxed and rehydrated after baking, and citric acid buffer was used for antigen repair and 3% H_2_O_2_ and methanol were used to inactivate endogenous peroxidase. Goat serum blocking solution was used to block samples, and antibodies against IL-6 (1:300, Proteintech, Chicago, United States) were used to incubate sample at 4°C overnight. Next, the samples were incubated with horseradish enzyme-labeled sheep anti-rabbit IgG polymer (ZSGB-BIO, Beijing, China), followed by diaminobenzidine (DAB) staining, hematoxylin staining, and dehydration. The sections were sealed and photographed.

### Statistical analysis

Data are presented as mean ± standard deviation (S.D.). GraphPad Prism software was used to perform the data analysis and draw related charts. The data were evaluated using a t-test or one-way ANOVA. Statistical significance was set at P < 0.05.

## Data Availability

The datasets presented in this study can be found in online repositories. The names of the repository/repositories and accession number(s) can be found in the article/Supplementary Material.
