# Supplementary material for: IL-6 signaling is required for the development and regeneration of ear cartilage in microtia
Source: Front Cell Dev Biol. 2025 Jul 30;13:1625058. doi: 10.3389/fcell.2025.1625058 (PMC12343669; doi:10.3389/fcell.2025.1625058)
Supplement: Supplementary file 1 [file DataSheet1.zip › Supplementary Material 1.docx]

**Detailed explanation of bioinformatics analysis**

1. Differential Expression Analysis

We analysed genomic profiling of three normal ear cartilage tissue and six microtia ear cartilage tissue in GEO#GSE227119 datasets to identify differentially expressed genes (DEGs) of microtia. NormalizeBetweenArrays algorithm was used to conduct the normalization and correction of data. When the gene expression was changed more than twice and the P-value was less than 0.05, it was identified as the DEGs. Pheatmap and ggplot2 packages in the R project was used to draw the related figures of DEGs.

2. WGCNA analysis

The NormalizeBetweenArrays algorithm was used to correct and normalize the data, and 13975 genes were included for WGCNA analysis, and the WGCNA package in R software was used to conduct analysis. We clustered the samples and set a suitable soft threshold (13) to establish the scale-free network. We next constructed adjacency matrix and topological overlap matrix and identified the related modules using hierarchical clustering. In addition, the eigengene were calculated and similar modules were merged (abline = 0.25). The genes in the same module have high topological overlap similarity, which means that these genes are highly co-expressed. At last, we calculated and identified modules with significant clinical relevance.

3. GO and KEGG analysis

We used the several packages (including ggplot2, org.Hs.eg.db, enrichplot, circlize, RColorBrewer, ggpubr, dplyr, complexHeatmap and clusterProfiler) in R project to conduct GO and KEGG enrichment analysis in order to detect the pathway and biological function involved by key genes of microtia. GO analysis includes cell biological process (BP), component (CC) and molecular function (MF).

4. PPI network and identification of hubgenes

The protein-protein interaction relationships of these key genes were obtained through STRING website (<https://cn.string-db.org/>) and Cytoscape software was used to visualize the PPI networks. These key genes were scored by using the cytoHubba plugin of Cytoscape software. We identified the top 15 genes with the highest scores as hubgenes based on the number of adjacent nodes.

5. Identification of diagnostic biomarkers of microtia

Support Vector Machine-recursive feature elimination (SVM-RFE), a supervised machine-learning algorithm, was used to identify the diagnostic biomarkers with superior discriminative ability in microtia. The Recursive Feature Elimination algorithm can achieve the optimal combination of variables which maximize the performance of model. The e1071, kernlab and caret packages in R software was used for SVM-RFE analysis.

6. Single-cell and intercellular communication analysis

We detected the cellular landscape and transcriptome between six normal ear cartilage tissue and three microtia ear cartilage tissue based on the GEO#GSE179135 dataset. SingleR package was used to conduct single-cell transcriptomic analysis. We integrated the Seurat objects into the merged data, and used principal component analysis algorithm to reduce the dimensionality of the data. FindNeighbors and FindClusters were used to cluster cells, and DEGs of cell were identified through FindAllMarkers. The annotation of cells was preformed through Hematopoietic.RData, ImmuneCellExpressionData.Rdata, ImmGenData.Rdata and Human_All.RData. Sqjin/CellChat package was used to conduct the intercellular communication analysis. For establishing the ligand receptor crosstalk network, CellChatDB.human was used. The contribution of each ligand-receptor was calculated through NetAnalysis_Continuation. PlotGeneExpression was used to detect the result of the level of ligands and receptors in signalling pathways. Senders, receivers, intermediaries and influencers were identified by using NetAnalysis_TCentrality and netAnalysis_SignalingRole_Network.
