# Supplementary figures and images for: IL-6 signaling is required for the development and regeneration of ear cartilage in microtia
Source: Front Cell Dev Biol. 2025 Jul 30;13:1625058. doi: 10.3389/fcell.2025.1625058 (PMC12343669; doi:10.3389/fcell.2025.1625058)

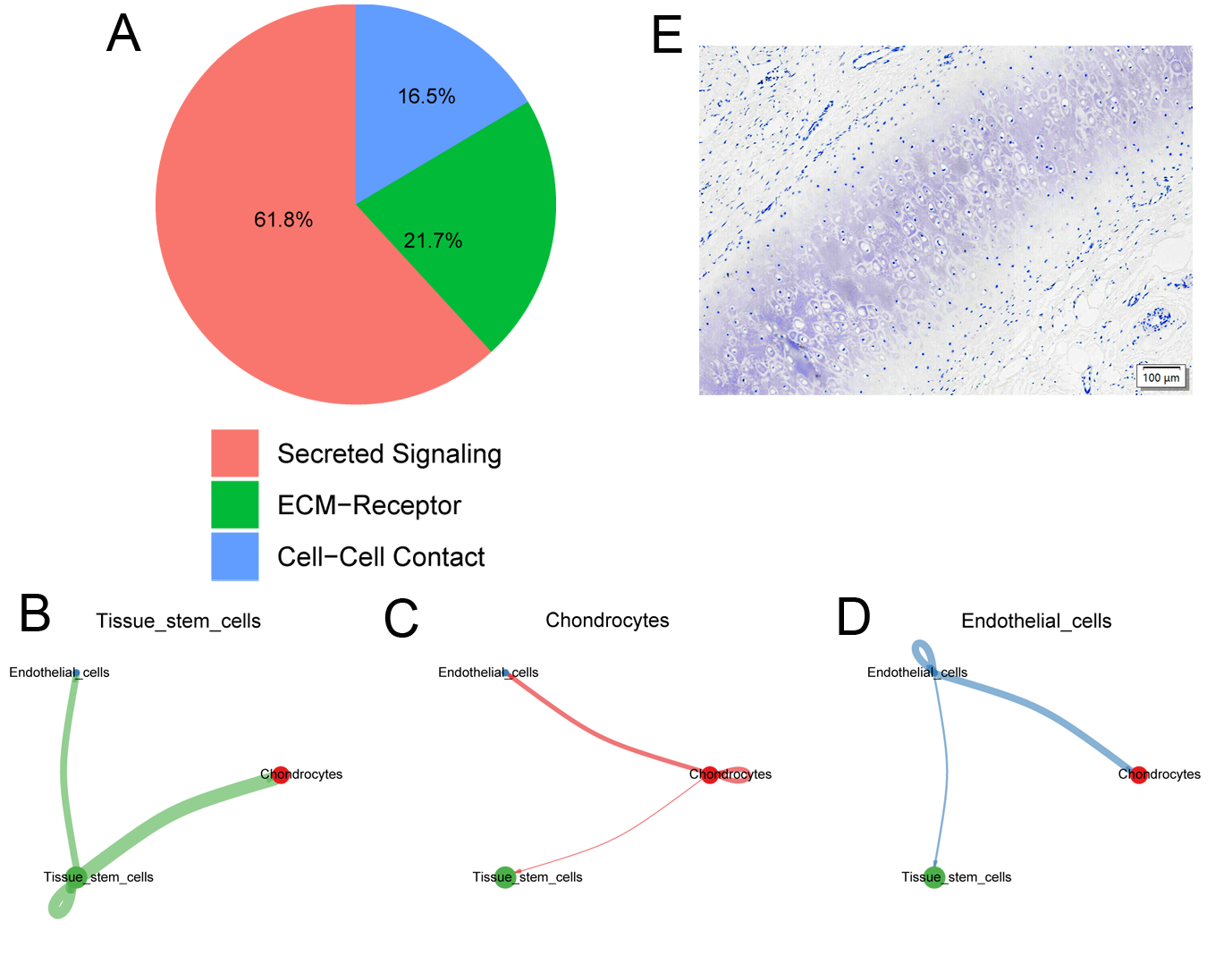

Supplement: Supplementary file 1 [file DataSheet1.zip › supfigure/sup╬┤▒Ω╠Γ-1.tif]

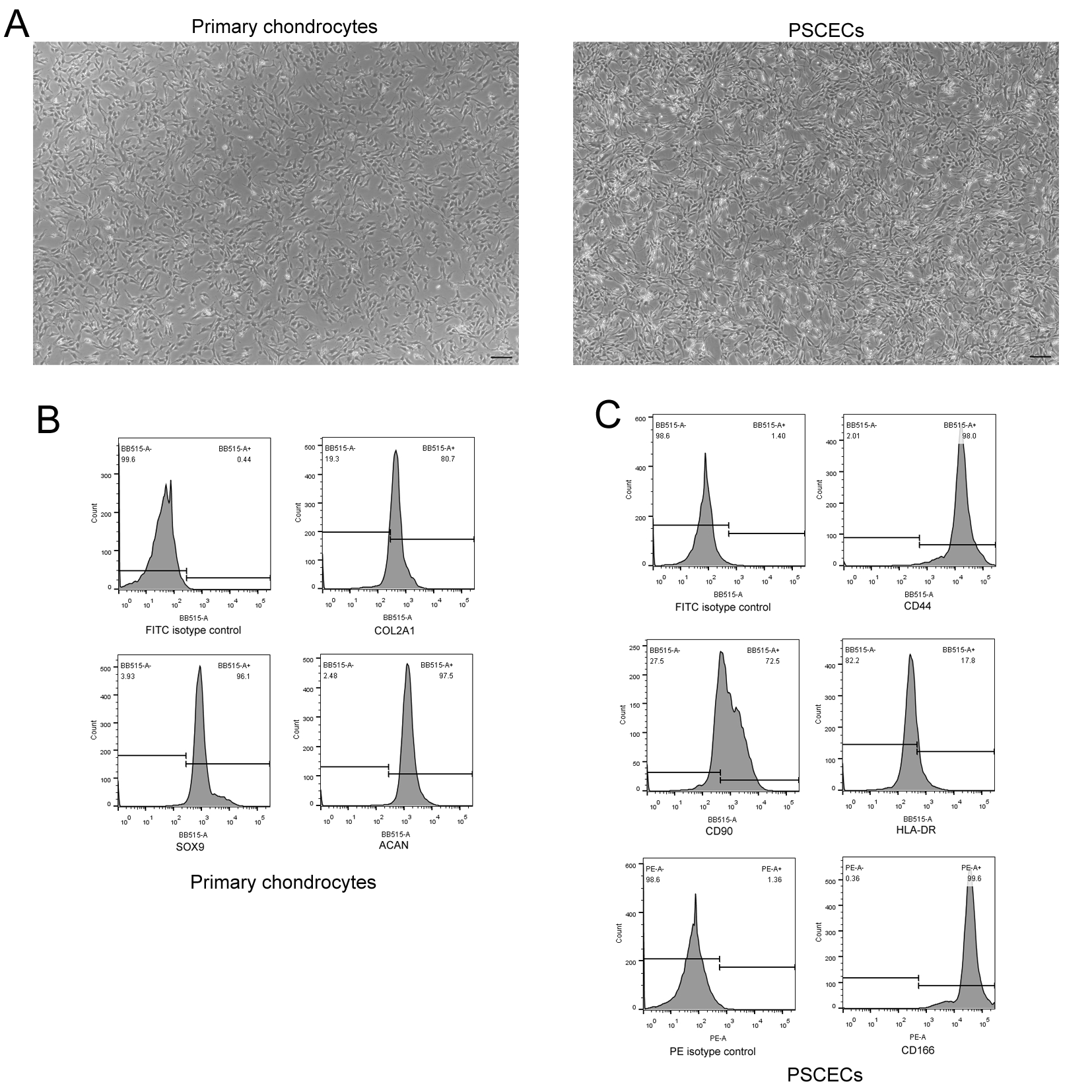

Supplement: Supplementary file 1 [file DataSheet1.zip › supfigure/sup╬┤▒Ω╠Γ-2.tif]

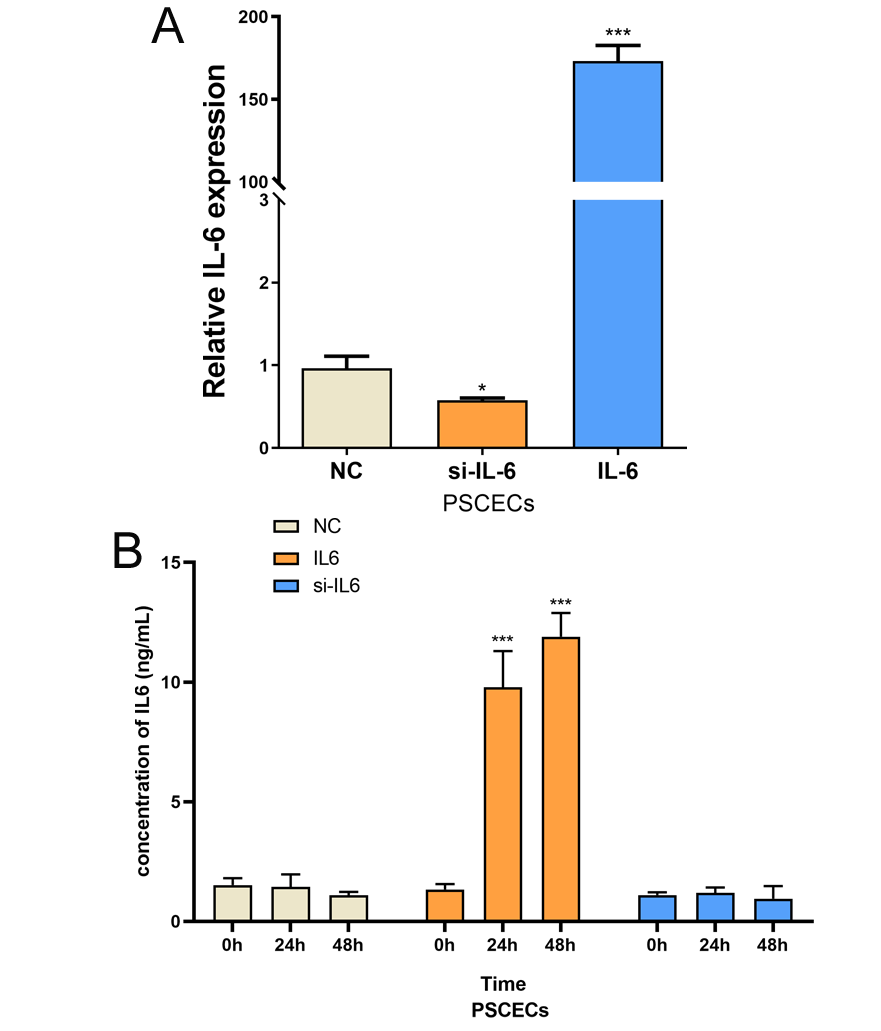

Supplement: Supplementary file 1 [file DataSheet1.zip › supfigure/sup╬┤▒Ω╠Γ-3.tif]
